# Expression of growth factors in colorectal carcinoma liver metastatic patients after partial hepatectomy: implications for a functional role in cell proliferation during liver regeneration

**DOI:** 10.1186/1476-5926-2-S1-S52

**Published:** 2004-01-14

**Authors:** Barbara Lukomska, Joanna Dluzniewska, Jerzy Polanski, Leszek Zajac

**Affiliations:** 1 Surgical Research and Transplantology Department, Medical Research Institute, Polish Academy of Sciences, Poland; 2 Department of Molecular Biology, Medical Research Institute, Polish Academy of Sciences, Poland; 3 2nd Department of Surgery, 2nd Medical Faculty, Medical University, Warsaw, Poland

## Introduction

Liver has a remarkable ability to replace lost cell mass. Surgical resection of hepatic lobes triggers hepatocyte replication. Normally hepatocytes have a quiescent, highly differentiated phenotype and rarely divide in adult humans [1,2]. However, their capacity to replicate is readily activated after liver resection or after toxic injury.

A number of studies have demonstrated the involvement of specific cytokines and growth factors in liver regeneration. The intact liver is relatively unresponsive to exogenous factors but partial hepatectomy provides some critical element that makes hepatocytes competent to fully respond to these substances [3,4]. Many growth factors play important roles in liver regeneration, most notably hepatocyte growth factor (HGF) and transforming growth factor a (TGF-alpha) [5,6]. HGF is an important multifunctional cytokine involved in liver repair after an injury. It acts as a motogen, a morphogen and a mitogen. Mesenchymal cells are responsible for the production of HGF whereas epithelial cells of various organs and tissues including the liver normally express HGF receptor cMET. In the liver HGF is produced by nonparenchymal cells especially perisinusoidal cells (PC), Kupffer cells (KC) and endothelial cells (EC). The expression of HGF mRNA during liver regeneration is also seen in mesenchymal cells in the lung and spleen and level of HGF increases in the blood after partial hepatectomy [7-9]. In contrast to HGF, which stimulates hepatocyte replication by a paracrine mechanism, TGF-alpha is an autocrine growth factor that is produced by hepatocytes and acts on these cells through binding the epidermal growth factor receptor (EGF-R) [10]. It is not known whether HGF and TGF-alpha have identical or complementary functions in hepatocyte replication.

Human liver regeneration is known to be influenced by the size of resection and also by the condition of the liver. Vascular complications and ischemic injury in hepatectomized patients could result in adequate regeneration, leading to hepatic insufficiency [11]. Moreover, regeneration of human liver is influenced by coexisting liver diseases. Clinical experience has shown that resection of diseased liver sometimes results in postoperative liver failure due to limited hepatic functional reserve. While considerable interest has been focused on the tumor recurrence in liver cancer patients who underwent partial hepatectomy (PH) no data have been reported regarding the regenerating process after PH in liver tumor-bearing patients. The growth factors released by cancer cells may possibly regulate the growth of other cells in paracrine manner.

The aim of the present study was initiated to determine the hepatocyte proliferation in relation to the expression of HGF and TGF-alpha in blood and liver tissue of patients with benign and malignant liver tumors after liver resection.

## Methods

### Patients

Twenty five consecutive patients undergoing partial hepatectomy for metachronous colorectal carcinoma (MCC) metastases (15 cases) and benign liver tumors (7 cases of angioma and 3 cases of cysts) were included in the study. All liver metastatic patients had currative surgical resection for primary colorectal carcinoma 4–36 months earlier and received chemotherapy. Informed consent was obtained from all patients and the trial was approved by the Medical Research Center Institute and Medical University of Warsaw Ethics Committees.

### Blood collection

Blood was collected from all patients before the operation, 30 min and 7 days after partial hepatectomy. The serum samples were stored at -20–C until they were used for HGF and TGF-alpha determination.

### Tissue samples

Surgical specimens were collected from: a) resected fragments of liver tissue remote from the tumor; b) tumor tissue; c) remnant liver, 30 min after hepatectomy; d) fine needle aspiration liver biopsy, 7 days after liver resection. Tissue samples were fixed in 10% buffered-formalin for 24 h before routine processing in paraffin embedding.

### Immunohistochemistry

Immunohistochemical detection of different antigens was based on avidin-biotin-peroxidase complex technique (LSAB+Perox rabbit, mouse, goat kit; DAKO, Glostrup). Monoclonal antibodies to proliferating cell nuclear antigen (PCNA) (NCL-PCNA, Novocastra), HGF (MAB294, R&D), cMET/HGF-R (NCL-c-MET, Novocastra), TGF-alpha (AF-239-NA, R&D) and EGF-R (sc-03, Santa Cruz) were used as primary antibodies. Briefly, sections were deparaffinized, endogenous peroxidase activity was blocked with 0.3% hydrogen peroxide in methanol for 30 min. For immunohistochemical detection of HGF, c-MET and EGF-R slides were immersed in citrate buffer (pH 6.0) and MV treated. For TGF-a staining, 0.01% protease digestion was performed for 10 min. Before incubation with primary antibody, the sections were blocked with normal swine serum, then the primary antibodies were applied and slides were incubated at 4 degrees C overnight. After washing, LSABox was applied. Visualization of peroxidase activity was achieved by mixture of 3.3' – DAB and hydrogen peroxide. Slides were counterstained with hematoxylin, mounted and evaluated under the light microscope. To avoid the relative human error in visual evaluation, we measured the intensity and the extent of immunostaining using a computer image analyzer (Micro Image, PC Sony 107, OLYMPUS, Japan) that analyze the image in numerical values.

### Quantitative determination of serum HGF and TGF-alpha level

Serum HGF and TGF-alpha level was determined with an enzyme-linked immunoabsorbent assay (ELISA) test kits (Quantikine; R&D Systems Europe, Ltd., UK (for HGF) and Oncogene MA, USA (for TGF-alpha) using commercially available reagents according to the manufacture's instructions. Briefly, serum samples were pipetted into the wells a microplate precoated with monoclonal antibody specific for HGF or TGF-alpha. After washing away any unbound substances, an enzyme-linked polyclonal antibody specific for HGF or TGF-alpha was added to the wells. Following a wash to remove the unbound antibody-enzyme reagent, a substrate solution was added to the wells and the intensity of the color was measured using a microplate reader (DYNATECH 5000). All measurements were performed in duplicate.

### Statistical analysis

Results are expressed as mean values – SD. The differences between two samples were defined as significant when *P *values by Mann-Whitney U test were less than 0.05.

## Results

### Clinical data

Fifteen patients with colorectal carcinoma liver metastases (8 men and 7 women, median age 62.2 – 7.3 {range 52–75 years}) and ten patients with benign liver tumors (3 men and 7 women, median age 48.4 – 4.3 {range 44–59 years}) were investigated. Preoperative liver parenchymal volume was equivalent in the two groups: malignant 1633.2 – 560.3 (range 977–1841) cm^3^; benign 1512.1 – 419.4 (range 986–2245) cm^3^. There were no statistically significant differences in the weight of resected liver tissue between patients with colorectal carcinoma metastases and those with benign liver tumors. Although the range of resected parenchymal weight in malignant and benign liver tumor patients was wide (462.5 – 271.5 {range, 120–890} g vs 356.8 – 217.6 {92–737} g, respectively) the distribution was comparable between two groups.

### Immunohistochemical analysis of PCNA in the liver

**Figure 1:**
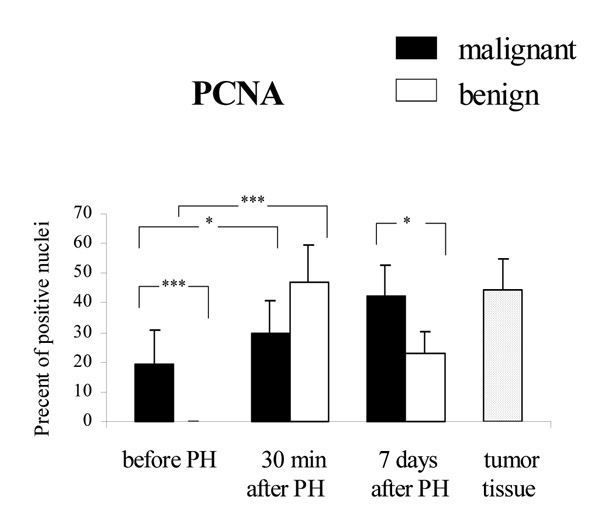
PCNA LI in the liver tissue of patients with metachronous carcinoma liver metastases (malignant) and benign liver tumors. Results are presented as the mean percentage of positive nuclei – SD (* p &lt; 0.05, ***p &lt; 0.001).

PCNA staining was examined in liver tissue sections taken from the resected organ and remnant liver 30 min and 7 days after hepatectomy. In cells showing positive PCNA reaction, the nucleus was stained either partially or entirely reddish brown. The ratio (%) of PCNA positive nuclei to all nuclei examined was calculated and represented the PCNA labeling index (PCNA LI) (Fig. 1). Positive PCNA immunostaining was observed in liver tissue of 60% patients 30 min after hepatectomy and in all patients 7 days after surgery in both groups of patients. PCNA LI was significantly higher in liver specimens of benign lesion patients taken 30 min after liver resection than in liver tissue before PH (47.1 – 12.2 vs 0, p &lt; .001). No difference in the LI was detected between liver tissue taken from malignant and benign liver tumor patients 30 min after hepatectomy, however the PCNA LI for colorectal liver metastatic patients was significantly higher than for benign tumor group 7 days after liver resection (42.4 – 10.2 vs 23.0 – 7.4, p &lt; .05). Interestingly, 9 of 15 patients with colorectal liver metastases revealed positive PCNA staining in liver cell nuclei of tissue samples taken from the resected lobes. In the group of patients with benign lesions no positive PCNA reaction was found. It was significant difference in the LI between malignant and benign liver tumor patients in liver tissue taken from the resected lobes (19.2 – 11.6 vs 0, p &lt; .001).

### Serum HGF level

**Figure 2:**
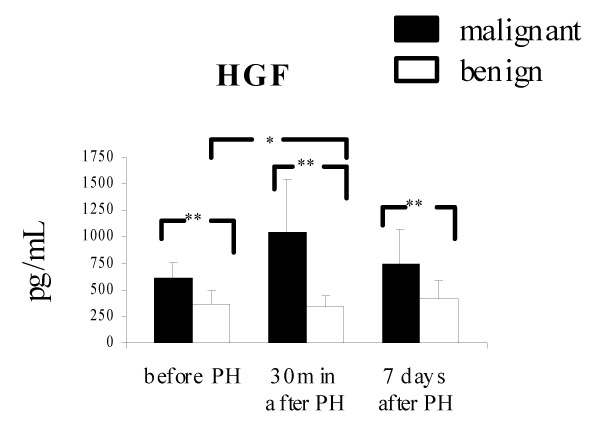
Changes in the serum HGF after partial hepatectomy in patients with metachronous colorectal carcinoma liver metastases (malignant) and benign liver tumors. Results are expressed as the mean value of pg/mL – SD (* p &lt; 0.05, ** p &lt; 0.01).

Mean circulating levels of HGF in benign and malignant liver tumors were within the reference range. There were no significant differences in the serum HGF level of benign liver tumor patients between the blood in the pre-operative period and blood taken 30 min and 7 days after partial hepatectomy (366.6 – 127.9 vs 343.9 – 99.4 vs 422.6 – 159.9 pg/mL, respectively). Serum levels of HGF in patients with metachronous colorectal liver metastases were significantly higher compared with those of patients with benign liver tumors (618.3 – 145.2 vs 366.6 – 127.9 pg/mL, p &lt; .001, before operation; 1045.2 – 494.0 vs 343.9 – 99.4 pg/mL, p &lt; .001, 30 min after partial hepatectomy; 750.5 – 326.1 vs 422.6 – 159.9 pg/mL, p &lt; .01, 7 days after partial hepatectomy, respectively. In malignant liver tumor patients serum HGF concentrations were significantly increased 30 min after partial hepatectomy compared with the pre-operative levels and returned to the pre-operative levels 7 days after operation (1045.2 – 494.1 vs 618.3 – 145.2 vs 750.5 – 326.1 pg/mL, p &lt; .05, respectively) (Fig. 2).

### HGF and c-MET/HGF-R expression in liver specimens

**Figure 3:**
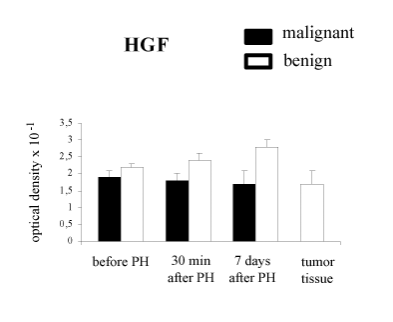
HGF expression in the liver tissue with metachronous colorectal carcinoma liver metastases (malignant) and benign liver tumors. Results are expressed as the mean optical density of positive immunostaining – SD.

HGF immunostaining was found in 80% (12 of 15) of liver tissue in colorectal liver metastatic patients and in all liver specimens taken from benign tumor patients. No difference in the intensity of HGF expression was detected between liver samples in malignant and benign tumor patients taken before and after hepatectomy (Fig. 3).

c-MET/HGF-R expression was found in 80% (12 of 15) of liver specimens isolated from resected lobes of colorectal liver metastatic patients and in all liver samples from benign tumor patients. The intensity of cMET staining in liver tissue before resection was higher in the benign than malignant group of patients (0.27 – 0.02 vs 0.18 – 0.02, p &lt; .05). No difference in the expression of c-MET was observed in liver tissue in both groups during liver regeneration.

### Serum TGF-alpha level

**Figure 4:**
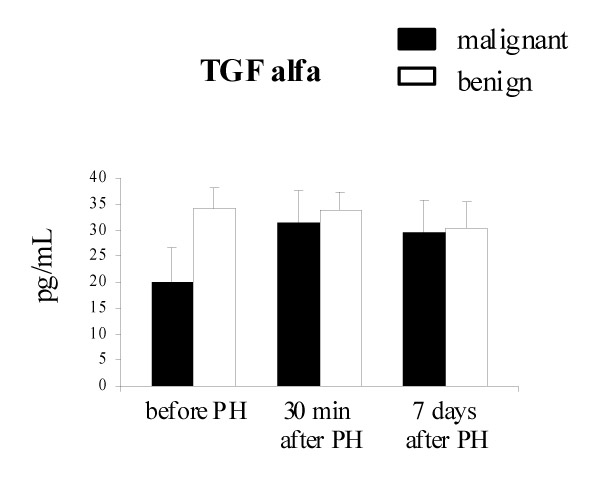
The level of circulating TGF-alpha in the serum of patients with metachronous colorectal carcinoma liver metastases (malignant) and benign tumors after PH. Results are expressed as the mean value of pg/mL – SD.

Serum level of TGF-alpha was very low in patients with malignant (20.1 – 6.5 pg/mL) and benign (34.2 – 3.8 pg/mL) liver tumor. No increase in TGF-alpha level was found in benign liver tumor patients after partial hepatectomy. Circulating TGF-alpha in peripheral blood of these patients was almost at the same level: 34.2 – 3.8 pg/mL before the operation, 33.7 – 3.7 pg/mL and 30.4 – 5.1 pg/mL 30 min and 7 days after PH, respectively. In patients with metachronous colorectal carcinoma serum concentration of TGF-alpha was significantly higher 30 min and 7 days after PH than before operation (31.5 – 6.0 and 29.5 – 6.2 vs 20.1 – 6.5 pg/mL, p &lt; 0.05, respectively) however, there was no difference in serum level of TGF-alpha between malignant and benign liver tumor patients after PH (Fig. 4).

### TGF-alpha and EGF-R/TGF-alpha R expression in liver specimens

**Figure 5:**
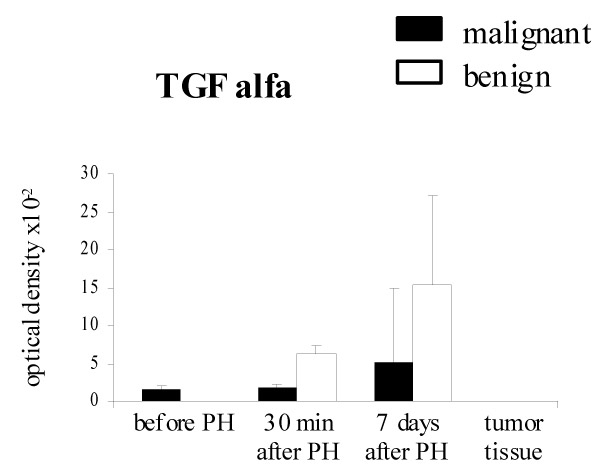
TGF-alpha expression in the liver tissue with metachronous colorectal carcinoma liver metastases (malignant) and benign liver tumors. Results are expressed as the mean optical density of positive immunostaining – SD.

TGF-alpha expression was detected in 26% (4 of 15) of liver samples taken from colorectal metastatic patients before and after hepatectomy. No TGF-alpha staining was observed in liver specimens taken from the resected lobes of benign tumor patients however, 20 (2 of 10) and 50% (5 of 10) liver samples displayed positive immunoreaction 30 min and 7 days after surgical resection, respectively. No marked difference in the intensity of TGF-alpha expression between both groups of patients during liver regeneration process was found (Fig. 5).

EGF-R was expressed in 86% (13 of 15) of liver specimens taken from malignant patients before and after hepatectomy and in all liver samples of benign liver tumor patients with various intensities. Lower level of EGF-R immunostaining was observed in liver tissue of colorectal liver metastatic patients than in patients with benign lesions before (0.20 – 0.04 vs 0.29 – 0.01, p &lt; .05) and 30 min after liver resection (0.17 – 0.03 vs 0.27 – 0.01, p &lt; .01) but no difference in the intensity of EGF-expression in liver tissue between both groups was found 7 days after hepatectomy.

### HGF, c/MET/HGF-R, TGF-alpha and EGF/TGF-alpha R in tumor tissue

HGF expression was found in 60% (9 of 15) of adenocarcinoma samples and the intensity of staining was similar to that observed in the liver tissue of malignant tumor patients. c-Met/HGF-R was detected in 33% (3 of 15) of tumor specimens. TGF-alpha immunostaining was not detected in tumor tissues but EGF-R expression was found in 53% (8 of 15) of tumors.

## Discussion

The capacity of the liver to restore major tissue loss within a few weeks is a unique process involving numerous interacting cells and a complex network of mediators. This capacity for liver regeneration is exploited clinically when partial hepatectomy is used in the treatment of disease. Clinical experience has shown that recovery after liver resection is related to the histopathological condition of the parenchyma, the risk of hepatic failure being higher when the organ is diseased [12,13]. Extensively studied in classical animals models, our knowledge of the cellular process that underlie liver regeneration in humans is incomplete. Liver regeneration following partial hepatectomy usually does not involve the activation of liver precursor oval cells. Instead liver mass is replenished by the proliferation of adult hepatocytes that may replicate *in vivo *under natural conditions. The multistep process of liver regeneration constitutes at least two critical phases: the transition of the quiescent hepatocytes into cell cycle (priming) and the progression beyond the restriction point in the G1 phase of the cycle.

The increase of liver mass most likely was caused by the rapid induction of hepatocyte proliferation as was shown by PCNA-positive hepatocytes. The PCNA level is very low in G_o _and early G_1 _phase of cell cycle and increases to a maximum in the S-phase when it becomes associated with the DNA replication sites. In our studies the number of patients showing positive PCNA staining in liver tissue was higher during regeneration process in both group 30 min and 7 days after liver resection. PCNA LI was significantly higher in liver tissue of patients with colorectal carcinoma liver metastases than in patients with benign tumor 7 days after partial hepatectomy (PH). Interestingly, PCNA LI in liver tissue taken from the resected lobes of malignant liver tumor patients was significantly higher than in liver of benign lesion patients. Normal liver tissue is negative for PCNA, thus PCNA positive immunostaining observed in liver specimens taken from resected lobes of metastatic patients argues for stimulation of hepatocyte proliferation by additional than liver cells' sources.

The correlation between liver cell proliferation and growth factors levels during liver regeneration in humans remains unclear. Although multiple factors rise in the plasma after PH it is likely that one of the key difference in blood between normal individuals and those subjected to liver resection is the increased amount of HGF. Hepatocyte growth factor could be produced in many organs or released from extracellular matrix of the liver [8]. The serum HGF level alone is not an indicator of liver regeneration but it modulates this process since total blood exchange with normal blood following partial hepatectomy reduces and delays liver regenerative activity in the early stage [14]. Levels of circulating HGF may vary due to enhanced production, decreased hepatic clearance or both because the liver is the major organ through which HGF is eliminated from the circulation [15]. Serum HGF level increased in association with hepatocellular dysfunction, hepatic necrosis and systemic inflammation [16,17]. In our studies we didn't notice any changes of HGF level in blood of liver benign tumor patients after PH however, the increase of serum HGF concentration was observed in colorectal carcinoma metastatic patients 30 min after liver resection. The amount of circulating HGF was significantly higher in patients with malignant liver tumors than in patients with benign lesions. Moreover, HGF level was higher in preoperative serum of patients with colorectal carcinoma liver metastases compared with those with benign tumors. This could be associated with the presence of adenocarcinoma. Patients with liver metastases showed slightly higher HGF concentrations in preoperative serum compared to that without liver metastases [18]. It has been reported previously that HGF is detected in human cancerous lesions of various organs [19-22]. In our study, HGF expression was found in colorectal liver metastases. This factor could be implicated in liver cell proliferation. It has been suggested that HGF plays a bifunctional role in invasive behavior of various tumors and also in the tissue repair and regeneration in reaction to tissue damage [23].

HGF is a potent stimulator of DNA synthesis in hepatocytes and interacts with other growth factors. Transforming growth factor (TGF-alpha) is another cytokine involved in hepatic regeneration [24,25]. TGF-alpha is speculated to interact with HGF in the induction of liver regeneration following partial hepatectomy, however it is two to three times less potent than HGF [26]. TGF-alpha appears to play a role at later times during liver regeneration. It is induced in hepatocytes within 3 hours after PH and rises to a peak between 12 and 24 hours [5]. Most if not all types of normal epithelial cells synthesize TGF-alpha. TGF-alpha can be produced by hepatocytes themselves thus inducing an autocrine loop. Usually only a very low level of TGF-alpha is detected in normal liver but it is higher in regenerating liver [27]. We did not observed an increase of TGF-alpha expression in liver samples taken 30 min and 7 days after PH. In our studies TGF-alpha expression was detected in liver metastases derived from colorectal carcinoma. Overexpression of TGF-alpha in liver metastases and primary carcinomas has been described by others [28]. TGF-alpha has a similar structure and function to epidermal growth factor (EGF) thus both TGF-alpha and EGF bind and activate the same receptor, EGF-R inducing mitogenic and motogenic response in many cell types. EGF-R expression observed in hepatocytes of regenerating liver may be involved in cross-talk between TGF-alpha /EGF-R pathway and the HGF/c-MET pathway inducing signal amplification as it was proposed by Jo et al [29]. In our studies we did not observe any difference in the intensity of HGF, TGF-alpha and their receptors staining in the liver tissue before and after PH. It might be due to metabolic fate of HGF/c-MET and TGF-alpha/EGF-R that are rapidly internalized and degraded as it was shown by *in vitro *experiments [30]. Therefore, growth factors expression alone is not sufficient to account in relation to liver cell proliferation in both groups of patients. Further studies at the gene level are needed. Messenger RNA (m-RNA) and protein levels in HGF and c-MET expression could be more reliable markers of liver growth.

In conclusions, our data demonstrated that the proliferation rate of liver cells was higher in patients with metachronous colorectal liver metastases than in patients with benign lesions, undergoing partial hepatectomy. It was correlated with increased level of circulating HGF. Human liver regeneration is known to be influenced by the size of resection and also by the condition of the liver. Since the resected volume of liver tissue was similar in the malignant and benign tumor groups other factors are important for sustaining proliferation of hepatocytes at higher level in patients with colorectal liver metastases than in patients with benign tumors. The mutual interactions between carcinoma cells and hepatocytes mediated by carcinoma-derived HGF may play a role in liver regeneration after partial hepatectomy.
